# Contribution of the epigenetic mark H3K27me3 to functional divergence after whole genome duplication in *Arabidopsis*

**DOI:** 10.1186/gb-2012-13-10-r94

**Published:** 2012-10-03

**Authors:** Lidija Berke, Gabino F Sanchez-Perez, Berend Snel

**Affiliations:** 1 Theoretical Biology and Bioinformatics, Department of Biology, Faculty of Science, Utrecht University, Padualaan 8, 3584 CH Utrecht, Netherlands; 2 Netherlands Consortium for Systems Biology, Science Park 904, 1098 XH Amsterdam, The Netherlands; 3 Applied Bioinformatics, PRI, Wageningen UR, Droevendaalsesteeg 1, 6708 PB Wageningen, Netherlands

## Abstract

**Background:**

Following gene duplication, retained paralogs undergo functional divergence, which is reflected in changes in DNA sequence and expression patterns. The extent of divergence is influenced by several factors, including protein function. We examine whether an epigenetic modification, trimethylation of histone H3 at lysine 27 (H3K27me3), could be a factor in the evolution of expression patterns after gene duplication. Whereas in animals this repressive mark for transcription is deposited on long regions of DNA, in plants its localization is gene-specific. Because of this and a well-annotated recent whole-genome duplication, *Arabidopsis thaliana *is uniquely suited for studying the potential association of H3K27me3 with the evolutionary fate of genes.

**Results:**

Paralogous pairs with H3K27me3 show the highest coding sequence divergence, which can be explained by their low expression levels. Interestingly, they also show the highest similarity in expression patterns and upstream regulatory regions, while paralogous pairs where only one gene is an H3K27me3 target show the highest divergence in expression patterns and upstream regulatory sequence. These trends in divergence of expression and upstream regions are especially pronounced for transcription factors.

**Conclusions:**

After duplication, a histone modification can be associated with a particular fate of paralogs: H3K27me3 is linked to lower expression divergence yet higher coding sequence divergence. Our results show that H3K27me3 constrains expression divergence after duplication. Moreover, its association with higher conservation of upstream regions provides a potential mechanism for the conserved H3K27me3 targeting of the paralogs.

## Background

Trimethylation of histone H3 at lysine 27 (H3K27me3) is a histone modification with an important role in regulation of gene expression [1]. It is generally associated with low expression levels and known as a repressive mark for transcription. Its function is conserved from animals to plants; however, there are several differences between the two kingdoms [2]. In animals, H3K27me3 marks long multi-gene regions of DNA while in plants it exhibits gene-specific positioning, starting at promoters and extending to the 3' end of the transcribed region, with a bias towards the 5' end of the gene [3]. It is deposited by Polycomb Repressive Complex 2 (PRC2) [4]. Interestingly, plants have several PRC2 complexes [5] that share some of their target genes while keeping a subset of targets unique for each complex [6]. It is not precisely known what directs PRC2 to its target genes in plants [7].

Functionally, H3K27me3 does not act as an all-on or all-off switch; instead, its placement is intricately regulated based on tissue type or environmental factors [8,9], similar to the gene-specific manner of regulation by transcription factors. For example, neighboring H3K27me3 target genes show no correlation in expression [3]. Genes with this epigenetic mark are functionally enriched for transcription factor activity, and are often involved in important processes in development [3,10-12]. In plants they are precisely regulated, showing tissue- or developmental stage-specific expression [3].

Little is known about the evolutionary processes shaping these expression patterns. In yeast and human, expression divergence between paralogs is correlated with coding sequence divergence [13,14], which is another measure of functional divergence. In plants, however, explaining expression divergence has proven to be a challenge. In *Arabidopsis thaliana*, old paralogs have diverged more in their expression patterns than newly duplicated genes, yet there is large variability within both groups [15]. It remains unresolved whether or not expression divergence correlates with the rate of coding sequence evolution [16-18]. Upstream regulatory sequence divergence is weakly correlated to expression divergence only for tandemly duplicated genes [16]. Additionally, the rate of expression divergence depends on protein function as well as the size and colinearity of the duplicated region [16,17], showing that a plethora of factors influence the rate of expression divergence between paralogs, and thereby their function.

In our work, we aim to see if H3K27me3 target genes show different trends in functional divergence after gene duplication than non-target genes. To achieve this we analyzed paralogs from the latest whole-genome duplication (WGD) in *A. thaliana*. The choice of model is warranted by the gene-specific positioning of H3K27me3 and a well-annotated recent WGD [15,19]. We determined divergence of coding sequences, upstream regulatory regions, and expression patterns. We show that H3K27me3 correlates with different rates of expression pattern divergence of *A. thaliana *paralogs. Paralogous pairs that are also H3K27me3 target genes exhibit a slower rate of function evolution as measured by expression pattern and regulatory sequence divergence. Paralogous pairs with only one H3K27me3 target gene, however, exhibit the most divergent expression patterns and regulatory sequences. On the other hand, divergence of coding sequence is the highest for H3K27me3 target paralogous pairs, and the lowest for non-target paralogs. This trend can be explained by expression levels [20,21]; namely, paralogs with H3K27me3 have lower expression and faster coding sequence evolution. The surprising trend in sequence divergence is especially prominent in transcription factors, the most abundant protein function among the H3K27me3 target genes. We show that, after a WGD, a histone modification is associated with slower divergence of expression patterns.

## Results

### Rate of expression divergence is associated with H3K27me3

To examine the correlation of H3K27me3 with the evolutionary fate of genes, we focused on paralogs arising from the most recent (3R or α) *A. thaliana *WGD. The advantage of limiting the analysis to a single WGD is that the resulting genes are of the same age and that the divergence time is thus equal for all of them, allowing us to simplify the analysis by eliminating time as a variable. Moreover, paralogs from large-scale duplications are more likely to be copied in their entirety, with intact coding and regulatory sequences. Additionally, because it is the most recent WGD, many paralogs are retained and relationships between them are well resolved. We used paralogous pairs as defined by Bowers and colleagues [19], a dataset consisting of 3,817 pairs.

Several genome-wide analyses have reported datasets with H3K27me3 target genes [3,22,23], most of them using entire *A. thaliana *seedlings despite the tissue-specific nature of the mark. These datasets are therefore information about an *'*average cell*' *in a seedling. We use them as a proxy for the entire plant: H3K27me3 is either present at a gene in any of the plant tissues or not present at all, simplifying H3K27me3 to a binary property of a gene.

To obtain a reliable set of target genes, we created a combined dataset consisting of genes reported in at least two out of three independent genome-wide experiments analyzing H3K27me3 localization in *A. thaliana *seedlings [3,22,23], totaling 6,338 genes (Figure s1 in Additional file 1; Additional file 2). As we consider H3K27me3 a binary property of a gene and compare pairs of paralogs, there are three possible outcomes resulting in three classes of paralogous pairs. The largest class, with 2,534 pairs, consists of paralogous pairs without H3K27me3, and is named *none*. In 18% of the cases one of the paralogs in the pair carries H3K27me3; these 652 pairs constitute the class *mixed*. The smallest class is *both*, consisting of 448 pairs (12%) (Additional file 3).

**Figure 1:**
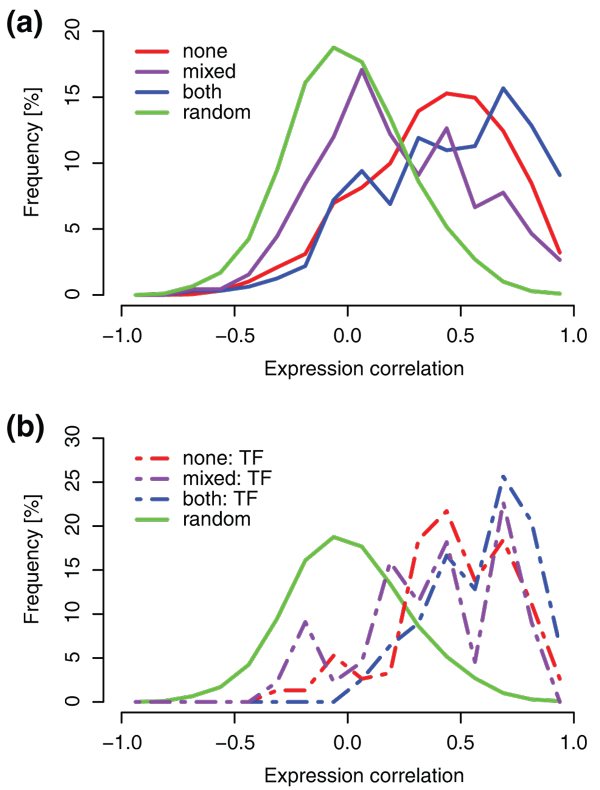
**Correlation of expression patterns of paralogous pairs**. **(a) **All paralogous pairs. **(b) **Paralogous pairs with transcription factor (TF) activity.

To determine if there is a relationship between the divergence of expression patterns of paralogs and mark presence, we calculated correlation in expression patterns for the three classes of paralogs. We obtained a number of publicly available microarrays from CORNET [24]. As H3K27me3 has been shown to play a role in developmental processes as well as in responses to environmental changes [3], the experiments range from various tissue types to different stress responses. The class with the highest expression correlation is *both*, with a median Pearson correlation coefficient of 0.49 (Figure 1a). It is followed by paralogous pairs without marks (*none*), with a median of 0.42. The two distributions are significantly different (Kolmogorov-Smirnov two-sided test, *P*-value 4.52e-5). Pairs in the class *mixed *show the highest divergence in expression with a distinctly lower median correlation of 0.16. This class is the closest to the random distribution (median 0.00), which was created by randomly combining genes into 10,000 pairs and calculating their expression correlation. *Mixed *is also significantly different from distributions where genes share the mark status (*P*-value 1.66e-15 for *both*, *P*-value <2.2e-16 for *none*). Remarkably, target genes of H3K27me3 show a common pattern in expression divergence: paralogs with H3K27me3 maintain more similar expression patterns.

We next wanted to resolve whether this surprising separation of class distributions is caused by the uneven separation of gene functions between the three classes. For example, transcription factors were reported to be the most enriched gene ontology category among the H3K27me3 target genes [3], and they are expected to be tightly regulated due to their crucial role in the regulatory network. While transcription factors from the 3R duplication retain more similar expression profiles than genes with other functions regardless of their class (Figure 1b; Figure s2 in Additional file 1), transcription factors in the class *both *(78 paralogous pairs) retain the most similar expression patterns, with a median expression correlation coefficient of 0.65. As in Figure 1a, it is followed by the class *none *(152 pairs; median 0.48) and the *mixed *class (44 pairs; median 0.41). Despite the small number of pairs in the distributions, the class *both *is significantly different from *none *(Kolmogorov-Smirnov two-sided test, *P*-value 1.1e-3) and the class *mixed *(*P*-value 1.2e-3); however, classes *none *and *mixed *are not significantly different to each other (*P*-value 0.09). Similar to other 3R paralogs, the transcription factor paralogs that are H3K27me3 target genes show more highly correlated expression patterns than the classes *none *and *mixed*. Thus, the difference between classes is also evident within a group of proteins with a similar function. Hence, proteins with transcription factor activity are not the main determinant for the trends we observed (Figure s2 in Additional file 1).

### Expression levels of H3K27me3 target genes explain coding sequence divergence but not expression divergence

**Figure 2:**
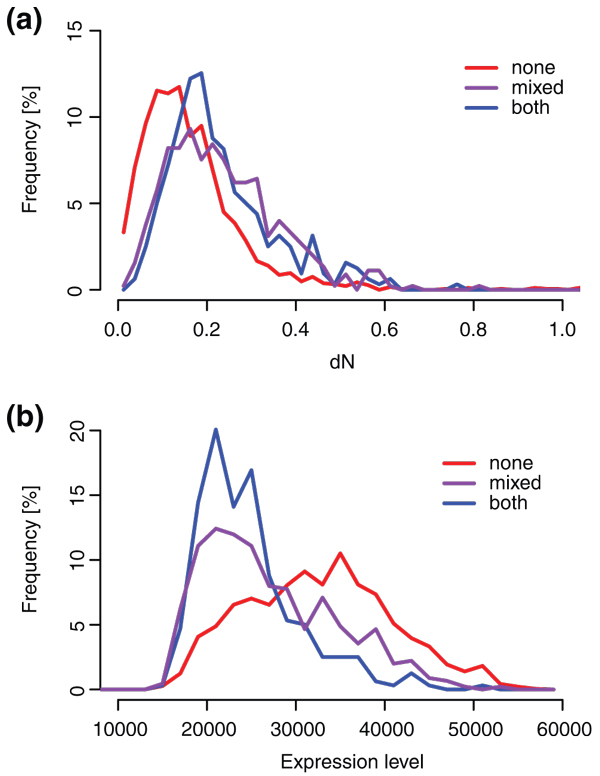
**Coding sequence divergence and gene expression levels**. **(a) **Distribution of Ka values. **(b) **Distribution of joint gene expression values for paralogous pairs.

Functional divergence of paralogs is not only estimated by analyzing differences in expression patterns, but also by determining differences in coding sequence. A positive relationship between the two measures has been observed in fungi and animals but is likely absent in plants [13,14,16-18]. For both reasons, we next wanted to determine if divergence of coding regions also shows separation of the distributions of the three classes, and if so, in what order. For every paralogous pair, we calculated the number of nonsynonymous substitutions per nonsynonymous site (dN). Two distributions are clearly separated (Figure 2a): genes in *none *tend to undergo the smallest number of synonymous substitutions (median dN 0.14). They are followed by paralogs with H3K27me3 (median dN 0.20). The two distributions are significantly different (Kolmogorov-Smirnov two-sided test, *P*-value <2.2e-16). *Mixed *has a median dN of 0.22 and a distribution different from that of *none *(*P*-value <2.2e-16) but not *both *(*P*-value 0.22). In contrast to expression divergence, where *mixed *shows the lowest conservation, also *both *shows low sequence conservation. This trend is also present for synonymous substitutions per synonymous site (dS) distributions, with class *both *showing the highest dS values (Figure s3 in Additional file 1). The opposite trends in coding sequence expression pattern divergence suggest not only lack of correlation between the two as reported previously [16-18] but for H3K27me3 target genes additionally a negative relationship between sequence and expression divergence. Sequence divergence cannot, therefore, explain the trends in expression divergence that we observed, and instead seems to be under the influence of different factors.

A possible factor for the faster sequence divergence of H3K27me3 target genes is their lower expression level compared to non-target genes [3]. Expression level has already been shown to be the main determinant of sequence divergence for a range of organisms, including *A. thaliana *[18,20,21,25-27]. Low sequence divergence of highly expressed proteins reflects selection against mistranslation and misfolding of the proteins, as these two outcomes present a high fitness cost for the cell. We thus hypothesized that the lower expression levels of H3K27me3 target genes could explain the trends in coding sequence divergence (Figure 2a). To test this, we summed the expression level of both paralogs in a pair across a number of microarray experiments [28]. Despite the noise that could be introduced by summing expression levels of two genes for each data point, the three distributions are significantly different (Figure 2b; Kolmogorov-Smirnov two-sided test, *P*-value <2.2e-16, <2.2e-16, and 5.4e-6 for the comparisons *both*-*none*, *mixed*-*none*, and *mixed*-*both*, respectively). As expected from previous results [3], paralogous pairs with H3K27me3 (class *both*) indeed have the lowest expression levels, and pairs that belong to *none *have the highest expression. With *mixed *placed much closer to *both *than *none*, the order of distributions is the same as for coding sequence divergence (Figure 2a). This corroborates the previously postulated link between coding sequence divergence and gene expression levels [18,20,21] and explains the sequence divergence in relation to mark status.

There is a possibility that low expression alone might lead to higher co-expression. In this case, the higher co-expression of paralogs in class *both *would be the result of their low expression. To address this confounding factor, we separated all paralogous pairs (regardless of which class they belong to) into five expression level categories (Figure s4 in Additional file 1), each containing 20% of the total number of paralogous pairs. Throughout the expression level categories, the most coexpressed class is *both*, followed by *none *and *mixed*. Furthermore, expression level is positively correlated to expression correlation (Figure s5 in Additional file 1); that is to say, lowly expressed genes tend to have low correlation. Thus, low expression is not a confounding factor for our main observation.

As the precise mechanism of H3K27me3 regulation is not known, we do not know whether low expression at a locus is a factor inducing trimethylation of K27 of that locus, or conversely, that low expression is simply the result of H3K27me3, which was directed to the locus by an unknown signal. We have shown that H3K27me3 is associated with a slower rate of expression pattern evolution, but cannot say whether it is also the cause.

### Regulatory sequence divergence of H3K27me3 targets corresponds to divergence in their expression patterns

**Figure 3:**
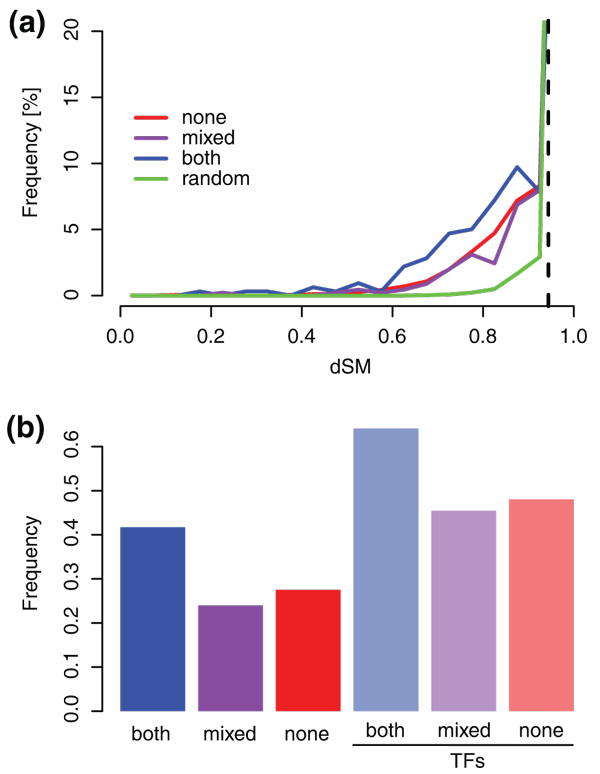
**Conservation of upstream regulatory regions as measured by dSM**. **(a) **Distribution of dSM scores between all paralogous pairs, according to H3K27me3. The dashed vertical line shows the dSM value at the fifth percentile of the random pairs (0.94). **(b) **Frequency of paralogous pairs with dSM lower than the fifth percentile cutoff.

Different regulatory mechanisms come together to shape gene expression patterns; while our focus is epigenetic modifications, transcription factors binding short DNA elements have a more direct effect on transcription. To see if paralogs with H3K27me3, which have more conserved expression patterns, also show more conserved upstream regulatory regions, we compared 500 bp upstream regions of paralogs. We used SharMot [29] to calculate the shared motif divergence score (dSM), which ranges from 0, for identical sequences, to 1, which means no similarity between the two sequences (Additional file 4). The dSM score was also calculated for 10,000 randomly combined pairs. We consider dSM values that are more similar than the 5% most similar randomly combined upstream regions (dSM = 0.94; Figure 3) to be indicative of conserved regulatory sites. We used this 5% cutoff to determine the optimal minimal length of the conserved upstream sequences (18 bp), and promoter length (500 bp). Shorter minimal length of conserved upstream sequences and longer promoter dramatically increase the number of false positives (determined by the number of hits in randomly combined pairs) in comparison to the number of all found conserved sequences (determined by number of hits in paralogous pairs).

The most similar upstream regions are those of class *both *(41% of all pairs), followed by *none *(26%) and *mixed *(23%) (Figure 3a). Transcription factors show even higher similarity: 63%, 47% and 45% of pairs, respectively, have significantly similar upstream regions (Figure 3b). The difference between *both *and *mixed*, and *both *and *none *is statistically significant (two-sample test for equality of proportions with continuity correction; *P*-values 1.02e-7 and 1.88e-7, respectively). While the difference between transcription factor-only classes is not significant due to the low number of pairs, there is significant difference between all gene and transcription factor classes (*P*-value 0.0007 for *both*, 0.0015 for *mixed *and 4.58e-8 for *none*).

Notably, the number of conserved upstream regulatory sequences is likely even higher as we report conserved sequences of promoters of 500 bp in length. Freeling and colleagues [30] examined the upstream regions of α WGD paralogs and found a number of genes rich in conserved upstream regions. They are significantly overrepresented in class *both *(*P*-value 3.37e-11, hypergeometric test) but not in *none *or *mixed *(*P*-value 1 and 0.56, respectively), in agreement with our findings. Paralogs with H3K27me3 have more conserved upstream regions, followed by *none *and *mixed*, which is comparable to the trend in expression pattern divergence, indicating that conserved upstream regions might hold the answer to different levels of expression pattern divergence.

## Discussion

In *A. thaliana*, the histone mark H3K27me3 localizes to individual genes [3], enabling us to follow the changes in each gene separately. Our first observation, higher sequence divergence of H3K27me3 target genes (Figure 1), can be explained by their lower expression levels, a correlation that has been reported previously [26]. More importantly, our analysis reveals a relationship between H3K27me3 target genes and conservation of expression patterns (Figure 2). We exclude low expression value as a confounding factor for our observation (Figure s4 in Additional file 1).

We aim to uncover an association of H3K27me3 target genes with a particular trend in their evolution, namely lower rate of expression divergence. We measured correlation in expression patterns over numerous different cell or tissue types and treatments to integrate regulatory information over many conditions. The H3K27me3 data were derived from seedlings, and represent a state in an average seedling cell. An average seedling cell is a statistical construct and might represent completely different levels of H3K27me3 in different seedling tissues. We therefore use the gene property *'*can be marked by H3K27me3*' *irrespective of the extent to which it is marked in the seedling (the fold-enrichment). This property is binary and allows a simple classification scheme of paralogs to see if they differ in a variety of aspects. In order to obtain a reliable definition of having H3K27me3 or not, we used an integration of datasets, as commonly used in integrative genomics [31,32], where at least two independent statistically significant calls are required to confirm that a gene is an H3K27me3 target.

Another epigenetic modification, DNA methylation of gene bodies, has been shown to correlate with other gene features in *A. thaliana*, specifically gene length and number of introns, as well as coding sequence divergence [33]. Epigenetic mechanisms have also been proposed for other observations, such as preferential deletion of paralogs from one homeolog, after a WGD in *A. thaliana *[34]. Our work, however, represents the first time that an association between a histone modification and establishment of expression patterns has been shown.

Based on our observations, we propose the following mechanism. Immediately after the duplication, the selection pressure is relaxed on both paralogs, and they can accumulate mutations and changes in regulation. If both genes keep H3K27me3, their expression patterns are likely to remain similar, possibly due to conserved elements in their upstream regulatory regions. For paralogous pairs without the mark, the expression pattern is mainly the result of transcription factors binding to their binding sites, which in turn also means lower upstream regulatory region conservation. Their expression patterns, though, are less similar than in *both *because H3K27me3 strongly represses transcription. Class *mixed*, on the other hand, shows highly divergent expression patterns: the paralog with H3K27me3 expression repression will be regulated by a different set of mechanisms and likely repressed in many tissues, and the resulting expression patterns will differ significantly between the two paralogs.

Paralogs in class *mixed *are also interesting because they show that H3K27me3 is not evolutionarily inert and that it has been possible to gain or lose the property of having H3K27me3 in the millions of years since the duplication event, and that the parental genomes contributing to the duplication event were necessarily not epigenetically identical (which is likely if the duplication event was an allotetraploidization). In our work, however, we do not aim to reconstruct the ancestral state of H3K27me3 in the parental genome. We analyze current associations between H3K27me3 target genes and their expression levels and correlation to their paralogs. Thus, the possibility that α WGD was an allotetraploidization event does not confound our results.

Due to a relatively long minimal length of the conserved upstream sequences at which we detect the strongest signal (18 bp), these sequences can hardly be attributed to a single transcription factor binding site. However, their function is uncertain: some might be *cis*-regulatory modules, a cluster of transcription factor binding sites. As hinted by higher conservation of upstream regulatory regions of paralogs in class *both*, other conserved upstream sequences might even have an H3K27me3-related function, such as RLE, a 50-bp element that has recently been found to be necessary for H3K27me3 deposition on LEC2 [TAIR: AT1G28300] [35]. More work will be needed to define the function of the conserved regions.

## Conclusions

H3K27me3 has an important role in regulation of gene expression in animals as well as in plants [1]. More so than animals, the plant *A. thaliana *is a uniquely suited model for our study because of gene-specific positioning of H3K27me3 and its recent WGD. We compared paralogs that emerged at the latest *A. thaliana *WGD and had the same amount of time to diverge. Because H3K27me3 is a tissue-specific epigenetic mark, and therefore not a permanent modification, it is remarkable that we observe such an effect.

Our first observation is that the rate of expression divergence differs between genes from different classes. Paralogs with H3K27me3 retain more similar expression patterns, while paralogous pairs with only one H3K27me3 target gene diverge the most. Paralogs in this class might show a higher divergence rate because H3K27me3 provides an additional and different layer of transcription regulation, together with transcription factors and other mechanisms. The difference in expression pattern divergence is the most pronounced for transcription factors. We show the same trends for conservation of upstream regulatory regions. In addition, pairs with H3K27me3 also show the highest coding sequence divergence, and are followed by class *mixed*, whereas pairs without H3K27me3 show the highest conservation of coding sequence. This is closely linked to expression levels, as H3K27me3 is a transcriptionally repressive mark and its target genes are expressed at lower levels.

To our knowledge, our work is the first to report an association between a histone modification and gene fate after duplication, and highlights the importance of epigenetics also as a factor in an evolutionary context.

## Materials and methods

### Datasets and general layout

We obtained paralogous pairs from the latest (3R, or α) *A. thaliana *whole-genome duplication [19], and three whole-genome analyses of genes carrying H3K27me3 (Figure s1 in Additional file 1) [3,22,23]. In order to increase the confidence in our combined dataset, we used only genes that appeared in at least two out of three H3K27me3 datasets (6,338 genes in total) as they were obtained using different methods (ChIP-chip, ChIP-seq, and ChIP-chip, respectively) and slightly differing plant material (10 to 14, 10, and 10 days after germination, respectively). Because several tissue types are represented in a seedling, the reported genes with H3K27me3 are a weighted average of the entire plant. As a consequence, we treat H3K27me3 as a binary property of a gene - that is, it is either present in any tissue or cell type, or not present at all.

The paralogous pairs were classified into three classes based on the number of genes in a pair that had H3K27me3: *both *(448 pairs), *mixed *(652 pairs), or *none *(2,534 pairs).

### Coding sequence similarity

To calculate coding sequence similarity, protein sequences and coding sequences (genome release version TAIR10) were obtained from TAIR [36]. For each paralogous pair we first aligned protein sequences using needle (EMBOSS 6.3.1) [37] (parameters: -gapopen 10.0 -gapextend 0.5) , and then performed protein-guided nucleotide alignment using backtrans from treebest 1.9.2 [38] (parameter: -t 0.5). From the resulting alignment we estimated dN and dS with codeml from PAML package v4.4 [39] using the Nei and Gojobori substitution model and the following parameters: noisy = 0; verbose = 2; runmode = -2; seqtype = 1; model = 0; NSsites = 0 ; icode = 0; fix_alpha = 0; fix_kappa = 0; RateAncestor = 0. Pairs with Ks > 5.0 were discarded because of unreliability of large Ks values, as were pairs with negative Ks values. These anomalies were attributed to changes in genome annotation between TAIR10 and the *A. thaliana *genome version used in [19]. The remaining 3,634 paralogous pairs (448 in *both*, 652 in *mixed*, 2,534 in *none*) were used in subsequent analysis.

### Expression

Expression correlation was obtained from microarray experiments (annotated as: PO:0009004: gametophyte, PO:0009008: organ, PO:0009002: plant cell, PO:0009008: sporophyte, PO:0009007: tissue, EXT:0000020: abiotic_stress_design, EXT:0000021: biotic_stress_design) from CORNET [24], comprising 2,231 slides (Additional file 5). They were normalized in R v2.10.1 using RMA from the affy package. Pearson correlation between two paralogs was calculated using a custom perl script. As ATH1 microarrays do not contain probes for all *A. thaliana *genes, and we only made use of unique probes (identifiers ending with _at), the number of pairs was reduced to 319 in class *both*, 451 in *mixed*, and 1,865 in *none*. Thus, the percentage of retained pairs was similar in all classes (71%, 69% and 74% of pairs, respectively).

The random distribution was obtained by randomly selecting 10,000 times two genes from the microarray, and calculating their expression correlation. We considered all genes annotated with the Gene Ontology term 'transcription factor activity' [GO:0003700] to be transcription factors.

For analysis of expression levels, the expression values were summed over all experiments for both genes in a paralogous pair. To calculate the linear regression model (Figure s5 in Additional file 1), the Pearson correlation coefficient (r) was transformed using ln ((1 + r)/(1 - r)), as has been described previously [14,13].

### Similarity of upstream regions

The similarity of 500 bp upstream regulatory sequences of paralogs (downloaded from TAIR [36], genome version TAIR10) was calculated using SharMot [29], parameter -l 18. Parameter -l determines the minimal length of the perfect stretch of matching nucleotides. To obtain a random distribution, we combined randomly selected genes into 10,000 pairs. Comparison with previously reported genes with conserved upstream regions [30] was performed using bigfoot pairs that also appear in Bowers *et al*.s dataset [19] and do not contain *'*_oa*' *in their identifiers.

## Abbreviations

H3K27me3: trimethylation of histone H3 at lysine 27; WGD: whole-genome duplication.

## Authors' contributions

LB, GFSP and BS conceived the study and its design, and LB performed all analyses. LB drafted the primary manuscript. All authors contributed to and approved the final manuscript for publication.

## Supplementary Material

Additional file 1 **Supplementary figures**. Click here for file

Additional file 2 **H3K27me3 target genes**. Click here for file

Additional file 3 **Paralogous pairs used in the analysis, and their properties**. Click here for file

Additional file 4 **Conserved upstream regions**. Click here for file

Additional file 5 **Microarrays used in the study**. Click here for file
