# Supplementary material for: Contribution of the epigenetic mark H3K27me3 to functional divergence after whole genome duplication in Arabidopsis
Source: Genome Biol. 2012 Oct 3;13(10):R94. doi: 10.1186/gb-2012-13-10-r94 (PMC3491422; doi:10.1186/gb-2012-13-10-r94)
Supplement: Additional file 1 — Supplementary figures. [file gb-2012-13-10-r94-S1.pdf]

Additional file 1

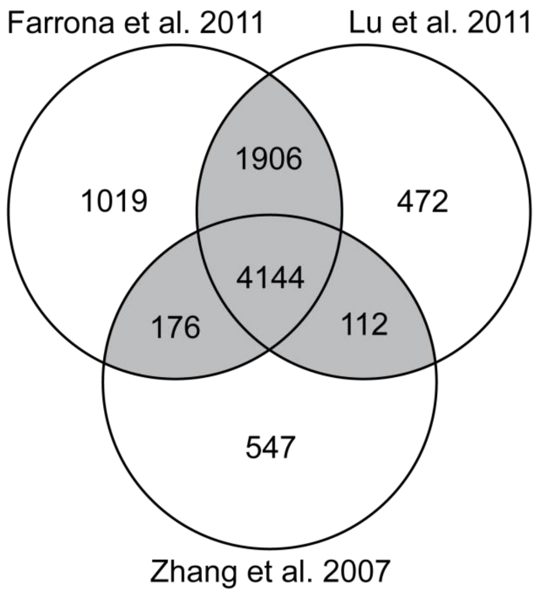

Suppl. figure 1: Comparison of three experiments reporting genes with H3K27me3.
Genes which were reported by at least two sources (in grey-shaded areas) were used for further analysis; a total of 6338 genes, or 75% of all reported genes.

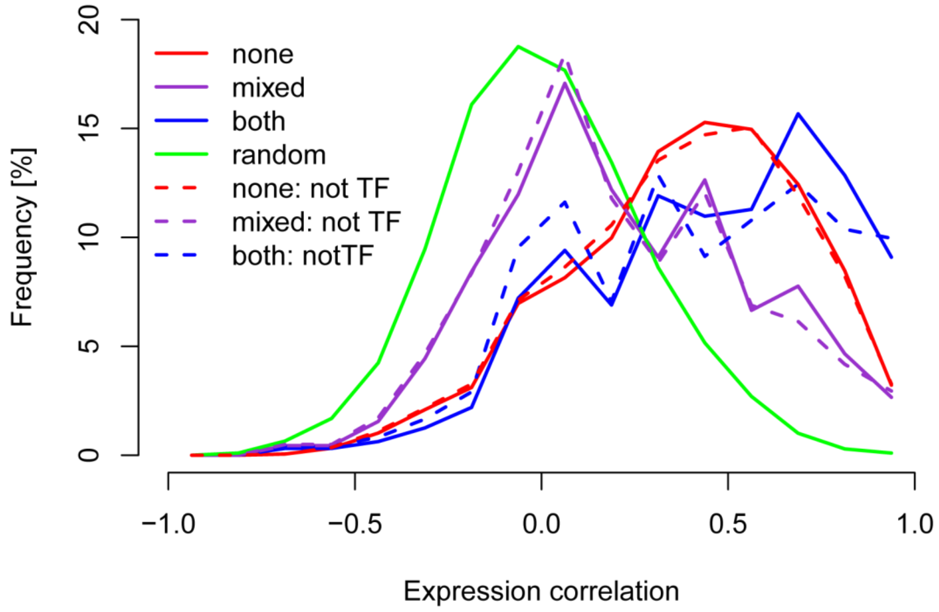

Suppl. figure 2: Comparison of expression pattern distributions.
All paralogous pairs (full lines) and paralogs not annotated as having transcription factor activity (dashed line).

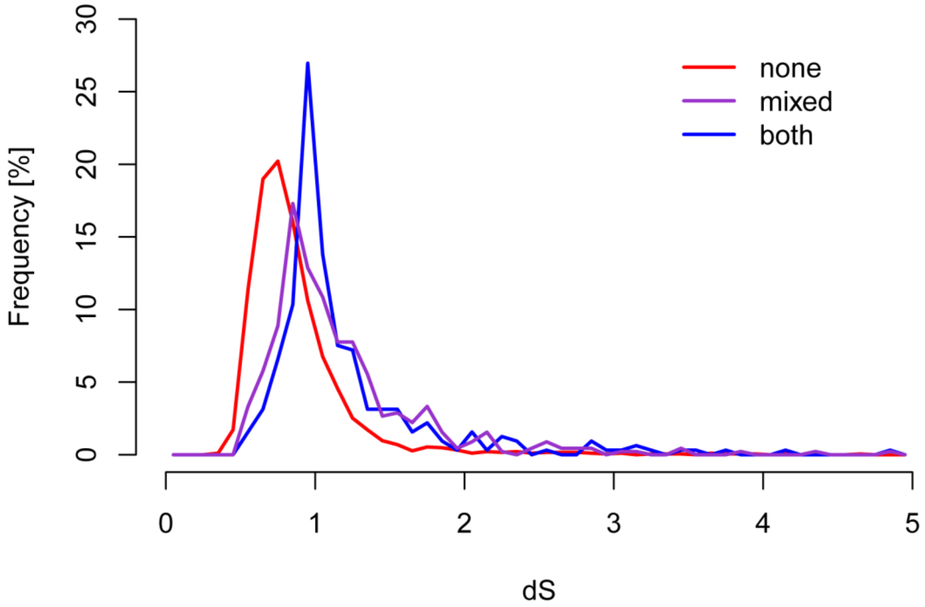

Suppl. figure 3: Distribution of Ka values.

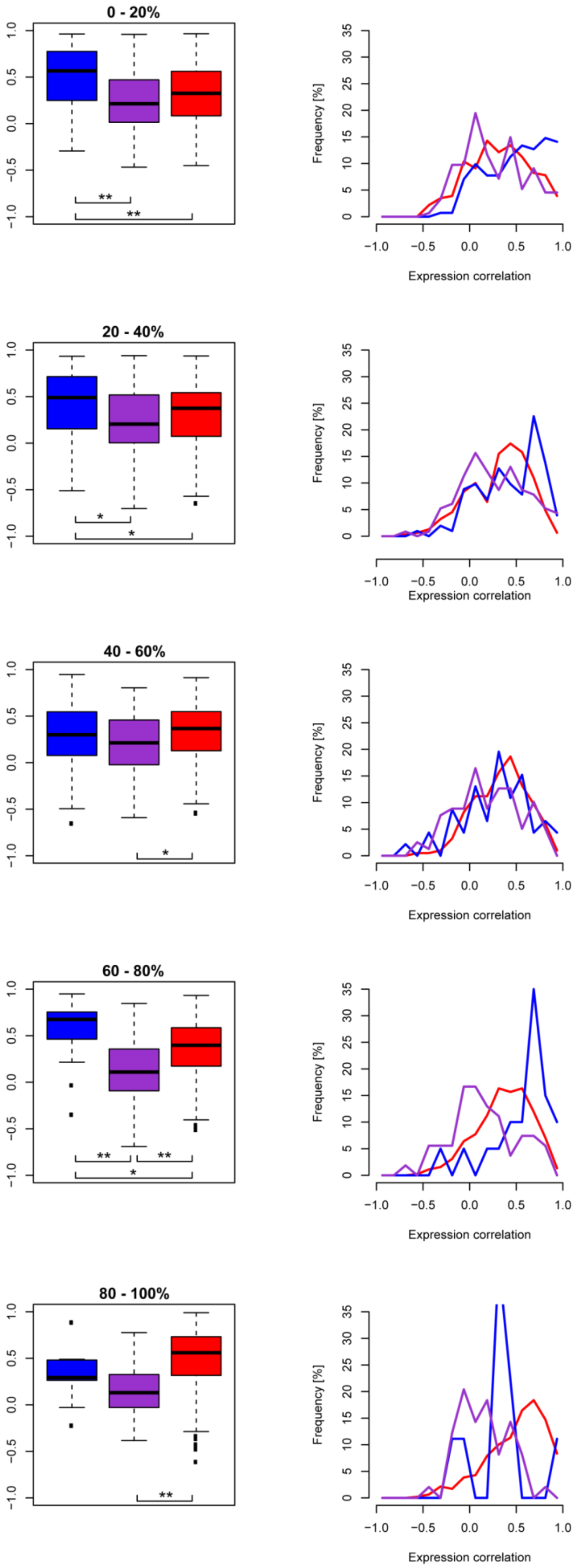
0 - 20%
20 - 40%
40 - 60%
60 - 80%
80 - 100%
Frequency [%]
Expression correlation

Suppl. figure 4: Analysis of expression correlation over five expression level categories.
All paralogs were split into 5 expression level categories, starting from the lowest 0-20% in top two figures. Statistical analysis was performed using Wilcoxon rank sum test (p < 1.0e-4 **, p < 1.0e-2 *). Note that number of paralogous pairs in the bottom two panels (with the most highly expressed 20% of paralogous pairs) is extremely low for class *both* (9 pairs in total).

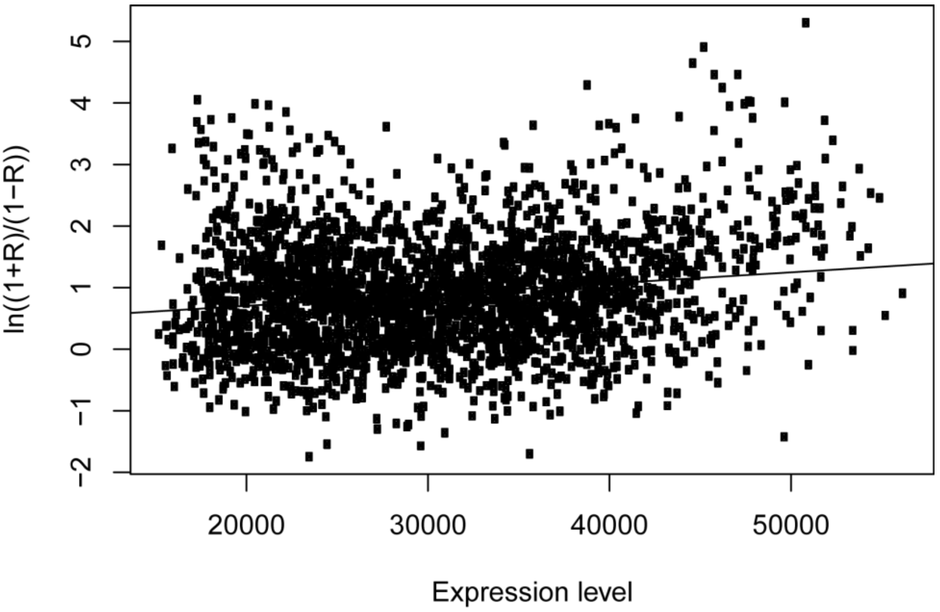

Suppl. figure 5: Relationship between expression level and expression correlation.
Expression correlation (R) was transformed using ln ((1+R)/(1-R)), and the relationship was analyzed using linear regression (p-value: < 2.2e-16).
